# Identification of novel small molecule inhibitors for solute carrier SGLT1 using proteochemometric modeling

**DOI:** 10.1186/s13321-019-0337-8

**Published:** 2019-02-14

**Authors:** Lindsey Burggraaff, Paul Oranje, Robin Gouka, Pieter van der Pijl, Marian Geldof, Herman W. T. van Vlijmen, Adriaan P. IJzerman, Gerard J. P. van Westen

**Affiliations:** 1 Division of Drug Discovery & Safety, Leiden Academic Centre for Drug Research, Leiden University, Einsteinweg 55, 2333 CC Leiden, The Netherlands; 2 Unilever Research & Development, Olivier van Noortlaan 120, 3133 AT Vlaardingen, The Netherlands; 3 Janssen Research & Development, Turnhoutseweg 30, 2340 Beerse, Belgium

**Keywords:** Sodium-dependent glucose co-transporter, Sodium-glucose linked transporter, SGLT1, Proteochemometrics, Molecular modeling, Machine learning, Cheminformatics

## Abstract

Sodium-dependent glucose co-transporter 1 (SGLT1) is a solute carrier responsible for active glucose absorption. SGLT1 is present in both the renal tubules and small intestine. In contrast, the closely related sodium-dependent glucose co-transporter 2 (SGLT2), a protein that is targeted in the treatment of diabetes type II, is only expressed in the renal tubules. Although dual inhibitors for both SGLT1 and SGLT2 have been developed, no drugs on the market are targeted at decreasing dietary glucose uptake by SGLT1 in the gastrointestinal tract. Here we aim at identifying SGLT1 inhibitors in silico by applying a machine learning approach that does not require structural information, which is absent for SGLT1. We applied proteochemometrics by implementation of compound- and protein-based information into random forest models. We obtained a predictive model with a sensitivity of 0.64 ± 0.06, specificity of 0.93 ± 0.01, positive predictive value of 0.47 ± 0.07, negative predictive value of 0.96 ± 0.01, and Matthews correlation coefficient of 0.49 ± 0.05. Subsequent to model training, we applied our model in virtual screening to identify novel SGLT1 inhibitors. Of the 77 tested compounds, 30 were experimentally confirmed for SGLT1-inhibiting activity in vitro, leading to a hit rate of 39% with activities in the low micromolar range. Moreover, the hit compounds included novel molecules, which is reflected by the low similarity of these compounds with the training set (< 0.3). Conclusively, proteochemometric modeling of SGLT1 is a viable strategy for identifying active small molecules. Therefore, this method may also be applied in detection of novel small molecules for other transporter proteins.

**Electronic supplementary material:**

The online version of this article (10.1186/s13321-019-0337-8) contains supplementary material, which is available to authorized users.

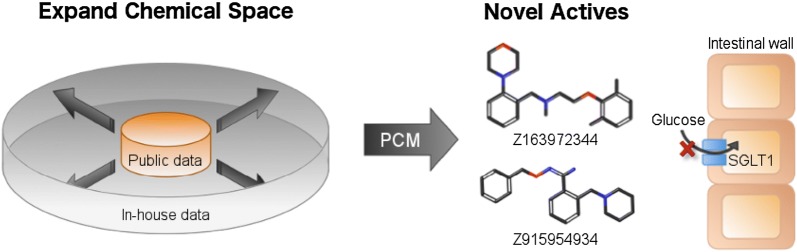

## Introduction

Sodium-dependent glucose co-transporters, or sodium-glucose linked transporters (SGLTs), are solute carriers (SLCs) that are responsible for glucose (re)absorption. SGLTs are members of the sodium-dependent transporters and are encoded by the SLC5A genes [1]. SGLTs are interesting targets in the treatment of diabetes mellitus, as their inhibition reduces the risk of hyperglycemia by decreasing glucose (re-)uptake [2]. In the human body two SGLT isoforms are involved in glucose transport: SGLT1 and SGLT2 [3]. Both SGLT1 and SGLT2 are expressed in the kidney, whereas SGLT1 is also expressed in the small intestine [4]. SGLT2 is a high capacity transporter responsible for 90% of glucose reuptake in the renal tubules and multiple compounds have been developed that inhibit this solute carrier [5, 6]. Furthermore, SGLT2 inhibition has been shown to decrease blood glucose levels in diabetes type 2 patients [7]. In contrast to SGLT2, SGLT1 is a low-capacity glucose transporter [1]. However, SGLT1 has a higher glucose affinity than SGLT2 and is additionally capable of transporting galactose [1]. Dual inhibitors blocking both SGLT1 and SGLT2 are currently in clinical development [8, 9]. In line with previous evidence we suggest that SGLT1 inhibition in the intestine will lower blood glucose levels as well [10, 11]. Compounds that do not penetrate the intestinal wall can achieve selective targeting of SGLT1 in the intestine, as they would not reach the renal tubules [12].

The complexity and the hydrophobic nature of transporter proteins make them challenging to crystalize. Crystal structures of transporters are scarce and binding locations of small molecules to these transporters are often unknown. For human SGLTs no protein structures are available negating the use of structure-based modeling techniques. However, the publicly available compound database ChEMBL includes ligand–protein binding information for multiple SGLTs [13–15], allowing the use of statistical modeling techniques such as quantitative structure–activity relationship analysis (QSAR) and proteochemometrics (PCM) [16]. These techniques, which make use of machine learning, do not require protein structural information and can therefore be applied in the context of SLCs. Although ligand-based pharmacophore modeling, QSAR, and PCM have only been applied to a few SLCs [17, 18], these techniques are well established on other drug targets including membrane proteins such as G protein-coupled receptors [19–21].

Unfortunately, the publicly available compound interaction data for SGLTs is limited from the point of chemical diversity as the major share of ligands are glycoside-like compounds and oxopyrrolidine-carboxamides. This limited chemical space hence restricts the applicability domain of QSAR and PCM models [22]. The applicability domain of computational models can be interpreted as the theoretical ensemble of molecular structures to which a model can be applied accurately. This domain is dependent on the model input and can therefore be quantified by similarity with the training molecules.

In the current work we show how we expanded the chemical space of SGLT inhibitors (using an in-house dataset [Oranje et al. manuscript in preparation]), and with that the applicability domain of our SGLT models. We constructed PCM models based on SGLT1 and its closest family members to predict compound activity for SGLT1. We successfully identified novel SGLT1 inhibitors that display low similarity towards the training set.

## Results and discussion

### SGLT chemical space

**Fig. 1:**
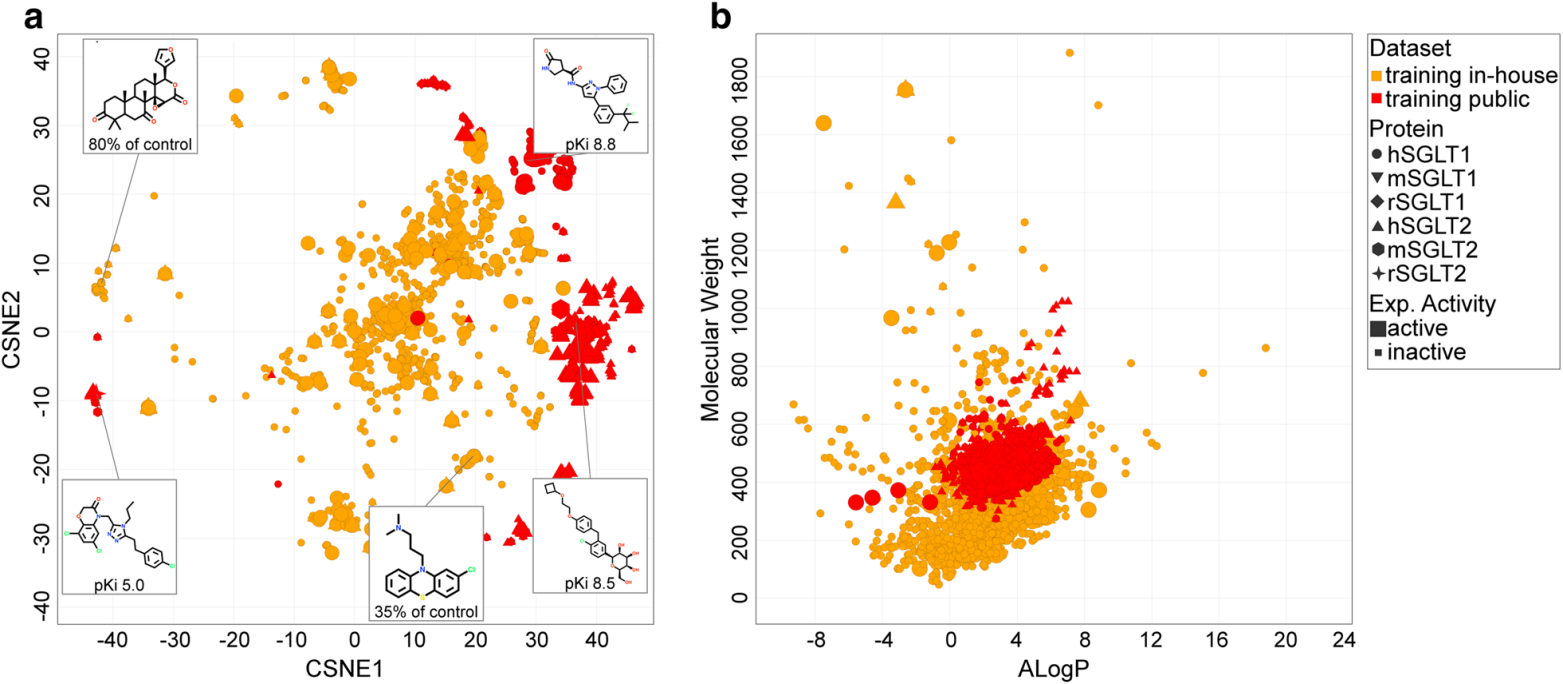
Chemical space of the public and in-house datasets. **a** The t-SNE shows molecular structure and affinity (pK_i_ for public data and % of (negative) control for in-house data) for representative hSGLT1 compounds. **b** Molecular weight and ALogP distribution of compounds in the training sets

A public dataset was created based on ChEMBL version 23 [13, 15] which includes the target protein human SGLT1 (hSGLT1), related protein human SGLT2 (hSGLT2), and multiple other SGLTs from different species. The public dataset encompassed 2063 data points and 1683 unique compounds, of which 886 compounds had measured hSGLT1 activities. Additionally, this set was supplemented with an in-house dataset of 2007 molecules previously screened for hSGLT1 and hSGLT2 inhibition [Oranje et al. manuscript in preparation]. This in-house dataset is based on the Spectrum Collection compound library [23] extended with compounds similar to primary screening hits and contained natural products and synthetic compounds. The data derived from ChEMBL was compared to the in-house dataset: the in-house dataset contained an additional 2005 hSGLT1 activities and 140 hSGLT2 activities, which were not present in the public dataset. The difference between the public and in-house dataset is graphically represented with t-Distributed Stochastic Neighbor Embedding (t-SNE) [24] (Fig. 1a, and Additional file 1: Figure S1 for graph color-coded on proteins). T-SNE was applied to decrease the high dimensionality of the datasets, making it possible to visualize them in 2D. The high dimensions are a consequence of the many descriptors that are used to describe the data, i.e. FCFP6 fingerprints. The t-SNE plot shows that the data derived for proteins similar to hSGLT1 extend the chemical space; many hSGLT2 compounds from the public domain are not tested on hSGLT1 and thus provide additional chemical information. The in-house and public datasets considerably differ from each other, with a slight overlap of only a few hSGLT1 and hSGLT2 public compounds with the in-house dataset. To further investigate the difference between the public and in-house dataset, the following physicochemical properties were considered: molecular weight, ALogP, and number of hydrogen bond donors and acceptors. The publicly available data represented mainly the drug-like space, following Lipinski’s rule of five, likely resulting from the fact that hSGLT2 is a drug target investigated by pharmaceutical companies [25]. Moreover, the public data mostly includes glycoside-like compounds and oxopyrrolidine-carboxamides. In contrast, the in-house dataset encompasses more diverse molecules and captures a wider value range for the physicochemical properties mentioned above. The molecular weight and ALogP are represented in Fig. 1b, where it is observed that these properties are more conserved for the public dataset than for the in-house dataset. Additionally, the number of hydrogen bond donors and acceptors is lower on average but more diverse in the in-house dataset (mean and standard deviation): public dataset hydrogen bond donor 3.6 ± 1.6 (vs 2.0 ± 2.6 for the in house set), hydrogen bond acceptor 6.3 ± 1.8 (vs 5.1 ± 4.1 for in the in house set). When screening for compounds to target hSGLT1 in the intestine, it is favorable to consider compounds that do not necessarily adhere to Lipinski’s rule of five, as it is preferred to minimize compound absorption from the gastrointestinal tract. Therefore, the in-house dataset contributes substantially to the applicability domain and relevant chemical space for the statistical SGLT model.

### Merging different datasets

To merge the public and in-house dataset the difference in activity units for both sets had to be resolved. The public dataset contains pChEMBL values, representing a standardized unit for affinity and potency values such as K_i_, IC_50_, EC_50_, and K_d_ [26]. The potency values in the in-house dataset were available as percentage activity compared to (negative) control at a concentration of 50 μM, which could not be converted into a pChEMBL value. Hence, binary classification models were chosen over regression.

**Fig. 2:**
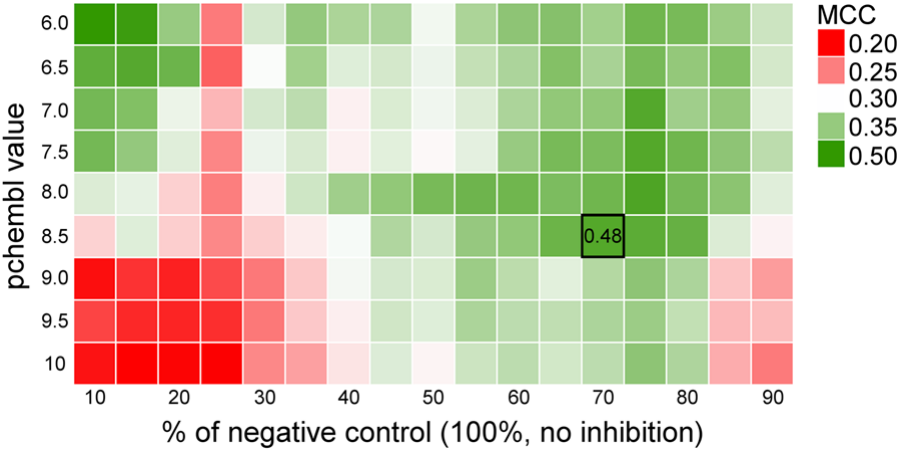
Activity threshold grid search. Searching the activity threshold grid for in-house (activity percentage compared to negative control) and public data (pChEMBL value). Model performance was measured using Matthews Correlation Coefficient (MCC), which was 0.48 for the final selected thresholds of < 70% for in-house data and pChEMBL > 8.5 for public data

Thresholds for compounds being ‘active’ were determined by grid searching cut-off values for both the public and in-house data. Activity thresholds along the grid were reviewed using hSGLT1 QSARs and external validation with a hold-out test set containing 30% of the in-house hSGLT1 data. The public domain compounds, which are mostly glycoside-like compounds and oxopyrrolidine-carboxamides, only describe a very conserved and small chemical space. However, the molecules of interest belonged to the same chemical space as the more diverse in-house compounds and therefore only compounds from the in-house set were used in validation. The activity threshold grid search showed that an activity threshold optimum for the in-house data was found at activity percentage of negative control < 70%, < 75%, and < 80% together with the threshold for public data set at pChEMBL > 8.5 (Fig. 2). In further models (see research workflow in Additional file 2: Figure S2) the activity threshold was set at activity < 70% for in-house data and pChEMBL > 8.5 for public data to achieve the best performance for predicting hSGLT1 active molecules in the chemical space of the in-house compounds. Although these activity thresholds are not similar toward each other (e.g. pChEMBL > 8.5 corresponds to an in-house threshold much lower than 70%), these thresholds were determined optimal for the aim, which is the identification of novel (weak) actives that are similar in chemical space as the in-house compounds. The performance of the QSAR benchmark model using the selected thresholds was: sensitivity 0.76, specificity 0.86, positive predictive value (PPV) 0.42, negative predictive value (NPV) 0.96, and Matthews correlation coefficient (MCC) 0.48.

### Proteochemometric modeling of hSGLT1

A PCM model was constructed using only public data to predict the inhibitory activity of compounds for hSGLT1. The performance of the model was tested on in-house data as these compounds represented the chemical space of interest. The model was validated using five test sets composed from in-house hSGLT1 data (5 × 20%). The mean performance of the public data model was very poor (mean with standard deviation): sensitivity 0.01 ± 0.01, specificity 0.98 ± 0.00, PPV 0.03 ± 0.06, NPV 0.91 ± 0.01, and MCC -0.03 ± 0.03 (Table 1). This demonstrates that with public data alone it was impossible to identify active compounds and the model defaulted to classification of all compounds as ‘inactive’. This behavior confirms the large differences in chemical space between the two sets as alluded to above.

**Table 1:** Model performance depends on datasets that are used in training

Model and validation	Training	Sensitivity	Specificity	PPV	NPV	MCC
QSAR (EV)	PD + IH	0.76	0.86	0.42	0.96	0.48
Public PCM (CV)	PD	0.01 ± 0.01	0.98 ± 0.00	0.03 ± 0.06	0.91 ± 0.01	− 0.03 ± 0.03
In-house PCM (CV)	IH	0.69 ± 0.07	0.89 ± 0.02	0.38 ± 0.06	0.97 ± 0.01	0.45 ± 0.05
Combined PCM (CV)	PD + IH	0.64 ± 0.06	0.93 ± 0.01	0.47 ± 0.07	0.96 ± 0.01	0.49 ± 0.05

*PD* public data, *IH* in-house data, *EV* external validation on 30% of data, *CV* fivefold cross validation on 20% of the data per iteration

Next, a PCM model was constructed based on the combined full data set consisting of all public and in-house data. To validate the performance of this model, fivefold cross-validation was applied with the same test sets as applied in validation of performance of the public data model: rotationally 20% of the in-house hSGLT1 data was used as holdout test set; the remaining 80% was used in training. In each case the test set contained compounds not available for training. This resulted in the following performance: sensitivity 0.64 ± 0.06, specificity 0.93 ± 0.01, PPV 0.47 ± 0.07, NPV 0.96 ± 0.01, and MCC 0.49 ± 0.05. Overall performance of this PCM model was regarded satisfactory for predictions of new compounds and was comparable with the QSAR benchmark model used for activity threshold determination previously.

Additionally the performance of models trained on in-house data only was tested to assess the effect of addition of public data. Public domain compounds contributed slightly to the predictive performance of the model in specificity, PPV, and MCC. This was observed by a minor decrease in performance upon removal of the public data from the training set: sensitivity 0.69 ± 0.07, specificity 0.89 ± 0.02, PPV 0.38 ± 0.06, NPV 0.97 ± 0.01, and MCC 0.45 ± 0.05. Although the difference in performances is not significant, it is remarkable that the number of false positives decreases considerably when public data is included in training, whereas the number of true positives is only slightly negatively affected: false positives 28 ± 6 versus 43 ± 6, true positives 24 ± 4 versus 26 ± 4 (with and without public data, respectively). Apparently, the public data by itself is not sufficient in predicting hSGLT1 activity in the chemical space of the in-house compounds but does add favorably to model performance when supplemented to the in-house dataset.

### Screening for hSGLT1 actives in a commercially available compound library

**Fig. 3:**
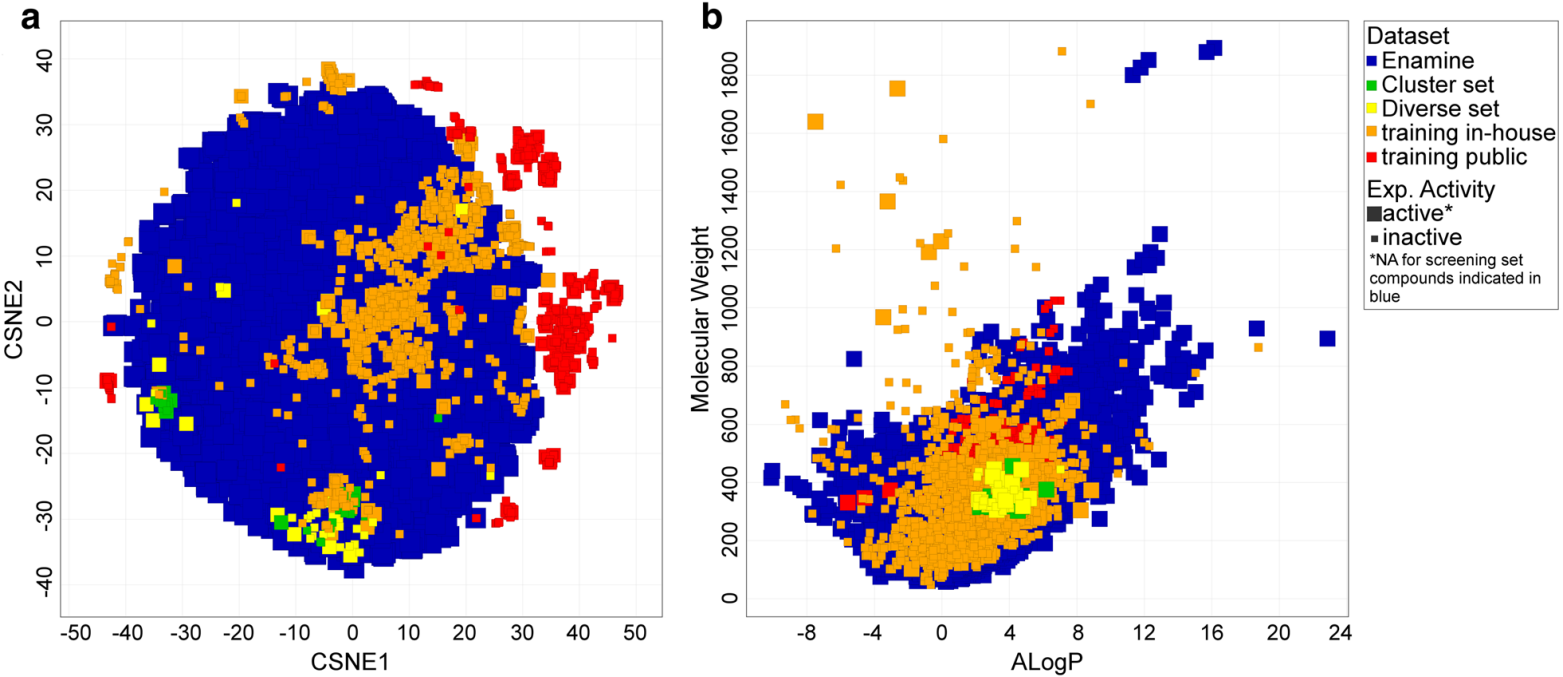
Chemical space of the selected compounds compared to the training and screening datasets. **a** The Diverse set (yellow) and Cluster set (green) are displayed compared to the training (orange and red) and Enamine screening set (blue). The Enamine set is represented by a random selection of 20,000 out of the total of 1,815,674 compounds (~ 1%) in the screening set to limit t-SNE calculation time. **b** The molecular weight and ALogP of the Diverse and Cluster set compared to the training and screening sets

The SGLT PCM model that was trained on public and in-house data was applied to a commercially available library. This library, the Enamine high-throughput screening (HTS) library, contains over 1.8 million compounds [27]. The library covers a wide diversity regarding molecular weight and ALogP values, and encompasses a vast chemical space (Fig. 3). With the PCM model (Additional file 3), an hSGLT1 activity prediction was assigned to all 1,815,674 compounds in the library (model training time was 103 s; the screening speed was approximately 132 s for 10,000 compounds). 155,275 compounds were predicted to be in the active class based on a predicted class probability of ≥ 0.5 (score, proportion of votes of the trees in the ensemble).

To increase confidence in the activity of compounds the screened set was pre-filtered by selecting compounds with a predicted class probability of ≥ 0.8 on a scale from 0 to 1. Here, a resulting score of 1 represents compounds predicted to be in the ‘active’ class, a score of 0 indicates that the compounds are predicted ‘inactive’; ascending scores indicate higher certainty of compounds belonging to the ‘active’ class. Additionally, compounds with molecular weight ≤ 300 were removed to exclude fragment-like compounds. The final filtered set contained 672 compounds.

**Fig. 4:**
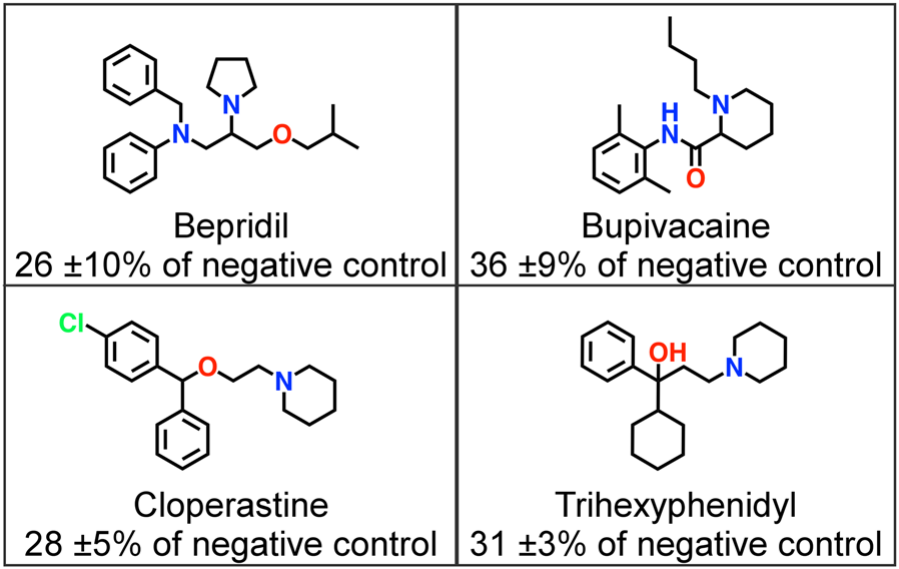
Reference hSGLT1 inhibitors for Cluster set and their inhibitory activity. Inhibitory activities (compared to negative control, where 100% is no inhibition) and chemical structures of four recently identified novel hSGLT1 inhibitors: bepridil, bupivacaine, cloperastine, and trihexyphenidyl

Based on the model predictions, 40 chemically diverse compounds predicted to be active were selected for experimental in vitro validation (‘Diverse set’). The compounds in this set were cluster centers resulting from clustering of the remaining predicted active compounds into 40 clusters. This diverse set was selected to increase the probability of detecting chemically novel hSGLT1 inhibitors. The selected compounds distributed widely through chemical space (Fig. 3 and Additional file 4: Figure S4), thus providing a challenging test for the SGLT PCM model. In addition to screening for novel hSGLT1 inhibitors, compounds were selected to expand the SAR around some recently identified hSGLT1 inhibitors from the in-house dataset [Oranje et al., manuscript in preparation]. Based on four hSGLT1 inhibitors (Fig. 4) 3 × 10 additional compounds were selected from the pre-filtered Enamine HTS set that were predicted to be active (with top ranking scores) and that resembled bepridil, bupivacaine, and cloperastine. Furthermore 7 compounds were selected resembling trihexyphenidyl (‘Cluster set’). These compounds were selected based on both model prediction (predicted class probability ≥ 0.8) and the highest similarity (Tanimoto, FCFP6) towards their known reference compound.

The total selection of 77 unique compounds was tested in vitro in cells expressing hSGLT1 in a single point measurement at a concentration of 50 μM. From the 40 diverse predicted hits that were assessed, 15 compounds were defined active as they displayed hSGLT1 inhibition in vitro with an activity reaching values below 70% compared to the negative control (100%: no inhibition) (Additional file 5: Data S5). From the 37 Cluster set compounds, an additional 15 compounds were confirmed to be active (Additional file 6: Data S6).

### Cytotoxicity of hSGLT1 actives

The potential cytotoxicity of the screening compounds (Diverse set and Cluster set) was investigated by analysis of secreted adenylate kinase (AK), a marker of cell wall integrity loss. Most compounds did not show any indication of cyotoxicity, however one active from the Diverse set displayed moderate impairment of the cell wall (Z1416510792: activity 43 ± 9%, cytotoxicity 25%). The cytotoxicity assay was limited by the available supernatant from the activity screen. Therefore not all compounds were measured in duplicate and cytotoxicity of one active from the Cluster set could not be determined (Z817504494: activity 45 ± 3%).

### Compound activity for hSGLT2

Both the Diverse set and Cluster set compounds were additionally measured for hSGLT2 inhibitory activity to assess their selectivity between the two transporters. The same cellular screening assay was performed as was used for hSGLT1 (single point measurement at a concentration of 50 μM). More actives were defined for hSGLT2 compared to hSGLT1 using the same activity threshold of 70% activity relative to negative control (100%: no inhibition): 22 actives in the Diverse set and 19 in the Cluster set. Almost all hSGLT1 actives showed activity for hSGLT2 with the possible exception of Z105569118, which only marginally surpassed the activity threshold for hSGLT2 (activity of hSGLT1 64 ± 4% and hSGLT2 76 ± 5%). No selective compounds were identified for hSGLT1, with 14% being the highest observed difference in inhibition (Z46160496: hSGLT1 41 ± 4% and hSGLT2 55 ± 2%). For hSGLT2 the biggest difference in inhibition was found for Z1318177320 that showed a difference of 39% (hSGLT1 93 ± 20% and hSGLT2 54 ± 0%).

### Hit compound analysis

The activities of the hit compounds of the Diverse and Cluster set were analyzed. The strongest inhibitors, Z163972344 and Z915954934, were derived from the Diverse set with activities of 24 ± 1% and 28 ± 4% (100%: no inhibition), respectively. Z163972344 has low similarity (0.27 based on Tanimoto FCFP6) with the training set, indicating that this is a truly novel inhibitor for hSGLT1. The average similarity of actives in the Diverse set compared to training was 0.33, with Z1416510792 being the active that is most similar to the compounds in the training set with a similarity score of 0.61 (this compound showed moderate AK secretion in the cytotoxicity assay).

For the Cluster set a total of 15 actives were validated for the four different clusters. The cloperastine cluster encompassed the most actives (60% actives), whereas the trihexyphenidyl and bepridil clusters contained the least actives with 29% and 30% actives, respectively. The bupivacaine cluster had an intermediate hit rate of 40%, which is comparable with the overall hit rate of the total Cluster set (41%). The variance in hit rates between the four clusters is also reflected in the similarity of compounds toward their cluster reference: the cloperastine and bupivacaine clusters contained the most similar compounds (average similarities towards cluster reference compound were 0.43 and 0.42, respectively); the trihexyphenidyl and bepridil clusters contained less similar compounds (0.35 and 0.31, respectively).

Although the cloperastine and bupivacaine clusters contained the most similar cluster members, no conclusive SAR could be determined. The cluster members displayed variations in methyl substituents, which showed an effect for two compounds in the bupivacaine cluster [Z46224544 (45 ± 10%) and Z2217101732 (74 ± 8%)]. This was however not observed for compounds in the cloperastine cluster: Z31367782 (36 ± 4%), Z31371621 (37 ± 3%), Z31367784 (43 ± 7%), and Z31370217 (45 ± 10%). The positions of the methyl substituents were too distinct to make solid conclusions on their relationship with compound activity.

In general, the novel active entities contain at least one aromatic ring and two hydrogen bond acceptors. Only two of the 30 actives did not adhere to Lipinski’s rule of five, with an ALogP of 5.2 and 6.2 for Z1844922248 (activity 49 ± 7%) and Z56906862 (activity 38 ± 5%), respectively.

### Aiming for specific targeting at the gastrointestinal tract

As mentioned in the Introduction, hSGLT1 inhibition at the intestinal wall is desired. Based on chemical structure and physicochemical properties the identified hit compounds will most likely be absorbed. However, it is suggested that modifications can be introduced to improve specific intestinal targeting. These alterations, such as a higher molecular weight, can prevent compounds from being absorbed or transported by the intestinal wall [28]. Intestinal SGLT1 blockers are expected to display less renal damage, which is an adverse effect observed for SGLT2 inhibitors [6]. Moreover, drug action restricted to the gastrointestinal tract also limits other off-target interactions, which were observed for the marketed SGLT2 inhibitor canagliflozin [29]. An example of a compound that was optimized for specific targeting at the gastrointestinal tract is LX2761, an inhibitor aimed at intestinal SGLT1 that decreased glucose uptake in mice [30, 31]. Although SGLT1 inhibition at the intestine may not compromise renal function, other adverse effects that can result from intestinal targeting need to be considered [32, 33].

### Indications for alternate binding modes

**Fig. 5:**
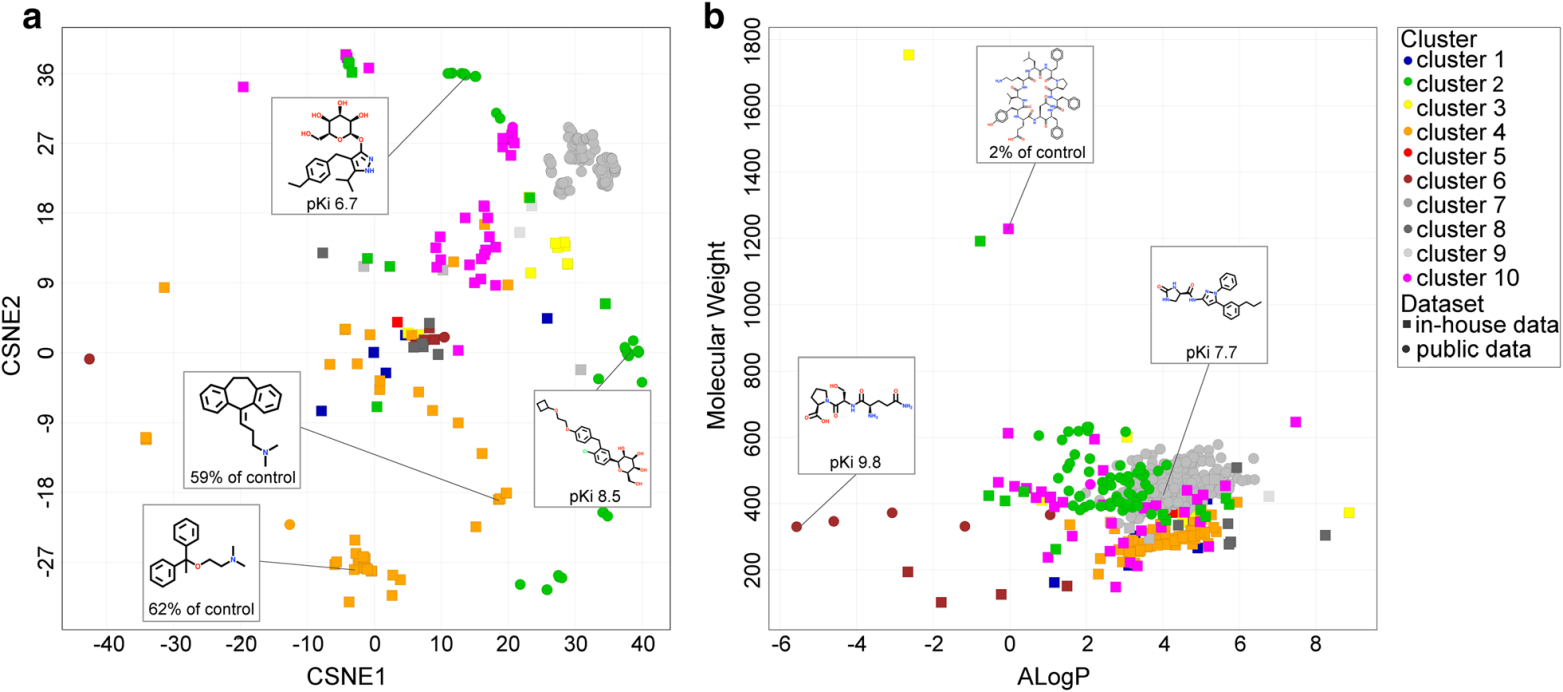
Clustering of hSGLT1 actives. Active hSGLT1 compounds in the training set clustered into ten chemical clusters (Tanimoto, FCFP6). Molecular structure and affinity (pK_i_ for public data and  % of (negative) control for in-house data) for representative cluster compounds are shown. In-house compounds with activity < 70% of (negative) control and public compounds with pChEMBL ≥ 6.5 were used in clustering. **a** t-SNE plot of the chemical clusters. **b** The molecular weight and ALogP distribution of compounds in the chemical clusters

Upon examination of our hSGLT1 actives, a large variety in chemical structure and physicochemical properties was observed. This indicates that different ligand types may bind to different sites on hSGLT1. It is speculated that the glycoside-like hSGLT1 inhibitors, which are represented well in the public compound domain, bind to the glucose binding site, whereas more chemically diverse hSGLT1 inhibitors are suggested to bind either there or elsewhere on the protein. The hSGLT1 actives were grouped into ten clusters. Here, the activity threshold for compounds from the public dataset was pChEMBL ≥ 6.5 to include all actives instead of only strong binders (pChEMBL > 8.5, which gave the best model performance). It was observed that the glycoside-like compounds cluster together in cluster 2 (Fig. 5). Furthermore, the oxopyrrolidine-carboxamide compounds, which are also present in the public domain, are gathered in cluster 7. Cluster 4 mainly holds in-house compounds and includes the anti-histamine drug moxastine and antidepressant amitriptyline besides cloperastine. The differences in chemical structure, molecular weight, and ALogP of the clusters substantiate the possible existence of multiple binding sites. As a further example, cluster 6 differs considerably in ALogP from the other clusters. This suggests that the compounds in this cluster bind to a more hydrophilic site. The cluster centers and distribution of molecular weight, ALogP, number of hydrogen bond donors, and number of hydrogen bond acceptors for all clusters are shown in Additional file 7: Figure S7. Additional pharmacological experiments, beyond the scope of this study, are warranted to further investigate the existence of multiple binding pockets in SGLT1. Attempts have been made to explore the binding sites of SGLT1 for substrates and inhibitor phloridzin [34, 35]. Although the SGLT structure of *vibrio parahaemolyticus* has been used to generate hypotheses on SGLT1 binding pockets, the lack of an hSGLT1 structure hampers the detection of potential allosteric binding pockets [36].

## Conclusions

We have demonstrated that PCM modeling is a viable method to identify novel inhibitors for solute carrier hSGLT1 and hence likely any solute carrier protein. A predictive SGLT model was built with a MCC value of 0.49 ± 0.05, estimated with fivefold cross-validation. With the optimized model a hit rate of 38% was achieved when it was applied to screen for diverse molecules (Diverse set). In parallel, the model was used to boost identification of actives with a given chemotype (Cluster set). Although additional active compounds were identified, the data was too ambiguous to gain insight into the SAR of hSGLT1 inhibitors.

Diversity was found within the in-house dataset and differences were observed between the in-house chemical space and that of the public dataset. Furthermore, the intrinsic variety in chemical structure of active compounds implies that there may be multiple binding sites at the transporter protein.

The novel identified inhibitors showed low similarity towards the training set and belong to the same chemical space of the in-house dataset, in contrast to the public dataset. Although the inhibitors were not optimized for specific drug delivery to the gastrointestinal tract, it is suggested that alterations (such as an increase in molecular weight and size) can make these inhibitors selective for intestinal hSGLT1.

## Methods

### Compounds and assay materials

DMEM-F12 (Biowest, Cat. No. L0092-500), DMEM (Lonza, BE12-604F/U1), Heat Inactivated Foetal Bovine Serum (HI-FBS, Biowest, Cat. No. S181H-500) and HBSS without Ca and Mg (HyClone, Cat. No. SH30588.01), DPBS (HyClone, Cat. No. SH30028.02), isopropanol (20,842.312), clear-bottom black 96 well plates (Greiner, Cat. No. 655090) and polypropylene 96-well plates (Nunc, Cat. No. 151193) were all obtained from VWR (Amsterdam, the Netherlands). TrypLE Express (Gibco, Cat. No. 12605010), geneticin (Gibco, Cat. No. 10131027), d-glucose free DMEM (Gibco, Cat. No. 11966025), water soluble probenecid (Invitrogen, Cat. No. P36400), 5000 U/mL penicillin–streptomycin (Gibco, Cat. No. 15070063) were all ordered from Thermo Fisher Scientific (Breda, the Netherlands). 1-NBD-Glucose was custom synthesized by Mercachem (Nijmegen, the Netherlands). Bovine serum albumin (Cat. No. A8806), poly-l-lysine hydrobromide mol. wt. 30,000–70,000 (Cat. No. P2636), cell culture grade DMSO (Cat. No. D2650) were all acquired from Sigma-Aldrich Chemie (Zwijndrecht, the Netherlands). The hSGLT1 cDNA cloned in the pCMV6-neo vector was purchased from Origene Technologies (Rockville, USA, Cat. No. SC119918). The hSGLT2 cDNA was custom synthesized and cloned into the pcDNA3.1 vector by Thermo Fisher Scientific (Breda, the Netherlands). The experimentally tested Enamine screening compounds were acquired from Enamine (Kyiv, Ukraine).

### Assay procedure

Two days in advance, CHO-hSGLT1 or CHO-hSGLT2 cells were seeded in maintenance medium (DMEM-F12 supplemented with 10% HI-FBS and 400 μg/mL geneticin) at 60,000 cells/well in clear-bottom black 96 well plates, pre-coated with 100 μg/mL poly-lysine. Cells were washed with 240 μL/well d-glucose free DMEM. Dilutions of test compounds and controls prepared in d-glucose free DMEM with 350 μM 1-NBd-Glucose, 0.3% BSA, and 2 mM probenecid were added at 90 μL/well and placed in a humidified incubator at 37 °C with 5% CO_2_ for 30 min. Subsequently cells were washed once with ice-cold DMEM-F12 and once with ice-cold HBSS, both at 240 μL/well. Finally, 1-NBd-Glucose was extracted from the cells with 100 μL/well isopropanol for 10 min at 600 rpm on an orbital shaker. Fluorescence was measured on a Flexstation 3 (Molecular Devices, San Jose, USA) with excitation at 445 nm, emission at 525 nm and cut off 515 nm. The uptake of 1-NBD-Glucose was normalized to the dynamic range between minimal inhibition (0.2% DMSO vehicle control) and maximal inhibition (100 μM phloridzin, > 100 × SGLT1/2 IC_50_). Phloridzin is a strong inhibitor of SGLT1 and SGLT2 and was used as 0% reference, with 100% being no inhibition. A concentration of 100 μM phloridzin was used to ensure full SGLT1/2 inhibition. The Z-factor for the controls was determined and only data with Z > 0.4 (average Z SGLT1 assays: 0.8 ± 0.1, average Z SGLT2 assays: 0.6 ± 0.1) was used [37].

### Cytotoxicity assay

The cytotoxicity of compounds was tested with the ToxiLight bioassay kit (Lonza, obtained from VWR, Amsterdam, The Netherlands) according to the supplier’s instructions. This non-destructive assay measures leakage of the enzyme AK from damaged cells into the CHO-hSGLT1/2 inhibition assay media, i.e. the degree of cytolysis. AK converts ADP into ATP and the enzyme luciferase subsequently catalyzes the formation of light from ATP and luciferin. Briefly, 20 mL of CHO-SGLT1/2 inhibition assay medium was added to 100 mL reconstituted AK detection reagent in white 96 wells Cellstar plates (Greiner bio-one, obtained from VWR, Amsterdam, The Netherlands) and incubated for 5 min at room temperature. Next, bioluminescence was measured on a FlexStation 3 Multi-Mode Microplate Reader (Molecular Devices, San Jose, USA) by 1 s integrated reading. Cytotoxicity was expressed as the percentage of bioluminescence of the 0.5% DMSO vehicle control which was set at 0%. The average cytotoxicity was calculated from biological replicates as indicated and average values > 20% were considered toxic (arbitrary threshold).

### Dataset

Publicly available data from ChEMBL (version 23) was extracted for human SGLT1 (accession: P13866), human SGLT2 (P31639), and related proteins human SGLT3 (Q9NY91), rat SGLT1 (P53790), rat SGLT2 (P53792), mouse SGLT1 (Q9QXI6), mouse SGLT2 (Q923I7), and mouse SGLT3 (Q8R479). The retrieved compounds were standardized by removing salts, keeping the largest fragment, standardizing stereoisomers, standardizing charges, deprotonating bases, protonating acids, and optimizing the 2D structure by correcting bond lengths and angles. Activity values with confidence score 7 and 9 were kept and duplicate activity values were discarded based on activity standard unit ranking: K_i_ > IC_50_ > EC_50_ > K_d_. For duplicate compounds with similar activity standard units (e.g. a compound with two K_i_ values), the average pChEMBL value was calculated.

An additional in-house dataset was provided by Unilever, Vlaardingen [Oranje et al., manuscript in preparation]. This dataset was based on the Spectrum Collection compound library (MicroSource Discovery Systems) extended with additional compounds that were similar to primary bioassay screening hits. This dataset consisted of compound activity data for hSGLT1 and hSGLT2. The activity was expressed as percentage 1-NBD-Glucose uptake compared to control at 50 μM, with control being the absence of inhibitor (= 100%). Molecular structures were standardized in the same manner as the public data. The final dataset (public and in-house datasets combined, no duplicates) encompassed 3686 unique compounds with 4208 derived activities, of which 2888 for hSGLT1.

### Compound descriptors

Compounds were described using 512 FCFP6 fingerprint bits and the following physicochemical properties: molecular weight, ALogP, number of hydrogen bond acceptors, number of hydrogen bond donors, number of rotatable bonds, number of bridge bonds, and number of aromatic rings. Fingerprints and physicochemical descriptors were calculated in Pipeline Pilot (version 16.1.0) [38].

### Protein descriptors

Protein sequences were aligned using whole sequence alignment in Clustal Omega (version 1.2.2) [39]. Subsequently the sequences were converted to protein descriptors using Z-scales [40]. The first three Z-scales were implemented as protein descriptor as these were shown to perform well in previous work [41]. These three Z-scales include information on residue lipophilicity, size, and polarity.

### Machine learning

Models were trained using the Random Forest R component in Pipeline Pilot (version 16.1.0). The number of trees was 500 and number of variables tried at each split was 38 (square root of the number of descriptors). Remaining settings were kept default.

### T-distributed stochastic neighbor embedding

T-SNE was calculated on FCFP6 fingerprint descriptors that were converted to 2024 bits. The t-SNE component in Pipeline Pilot (version 18.1.0) was used to perform tSNE. The derived t-SNE values are represented by two components: CSNE1 and CSNE2.

### Clustering of hSGLT1 actives to explore binding modes

hSGLT1 active compounds in the training set were clustered into ten clusters using the cluster molecules component in Pipeline Pilot (version 16.1.0). Compounds from the in-house set were included as ‘active’ when percentage of (negative) control was < 70%. Compounds from the public data set were termed ‘active’ when pChEMBL value ≥ 6.5.

### Computational hardware

Experiments were performed on a server running CentOS 6.9 equipped with a dual Xeon E-5 2630 v2 processor and 128 GB of RAM.

## Additional files

**Additional file 1.** T-SNE representation of the chemical space of the public and in-house datasets colored by species.

**Additional file 2.** Schematic overview of the experimental workflow of this study.

**Additional file 3.** Random Forest SGLT PCM model used for final predictions.

**Additional file 4.** T-SNE representation of actives and inactives of selected compounds compared to the training set.

**Additional file 5.** Bioactivities, cytotoxicities and Tanimoto similarities of the Diverse set.

**Additional file 6.** Bioactivities, cytotoxicities and Tanimoto similarities of the Cluster set.

**Additional file 7.** Cluster centers and distribution of physicochemical properties of hSGLT1 active compound clusters.

## Abbreviations

AK: adenylate kinase
HTS: high-throughput screening
MCC: Matthews correlation coefficient
NPV: negative predicted value
PCM: proteochemometrics
PPV: positive predicted value
QSAR: quantitative structure–activity relationship
SGLT1/2: sodium-dependent glucose co-transporter 1/2
t-SNE: t-distributed stochastic neighbor embedding
