# Supplementary figures and images for: Identification of novel small molecule inhibitors for solute carrier SGLT1 using proteochemometric modeling
Source: J Cheminform. 2019 Feb 14;11:15. doi: 10.1186/s13321-019-0337-8 (PMC6689890; doi:10.1186/s13321-019-0337-8)

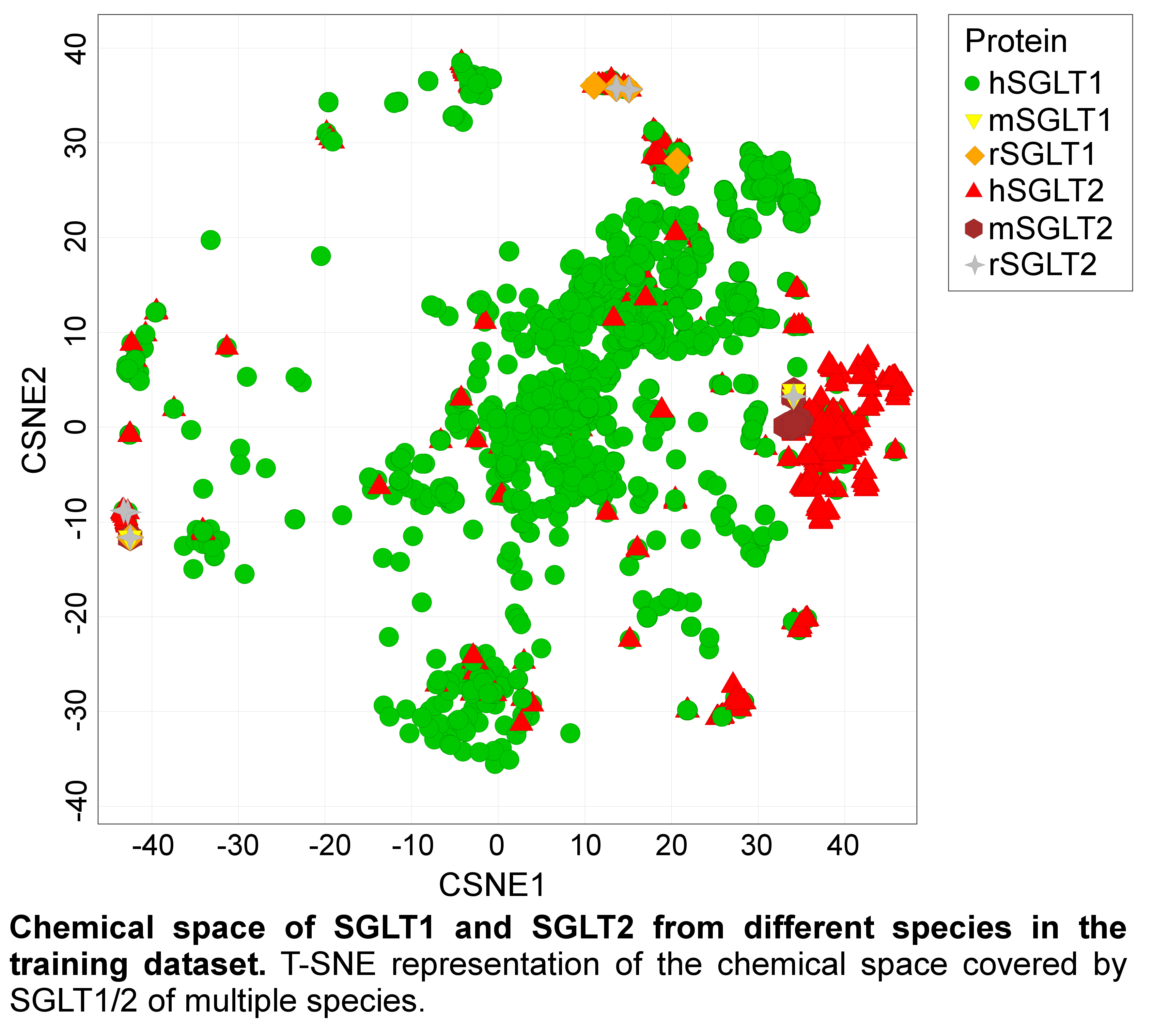

Supplement: Supplementary file 1 — Additional file 1. T-SNE representation of the chemical space of the public and in-house datasets colored by species. [file 13321_2019_337_MOESM1_ESM.png]

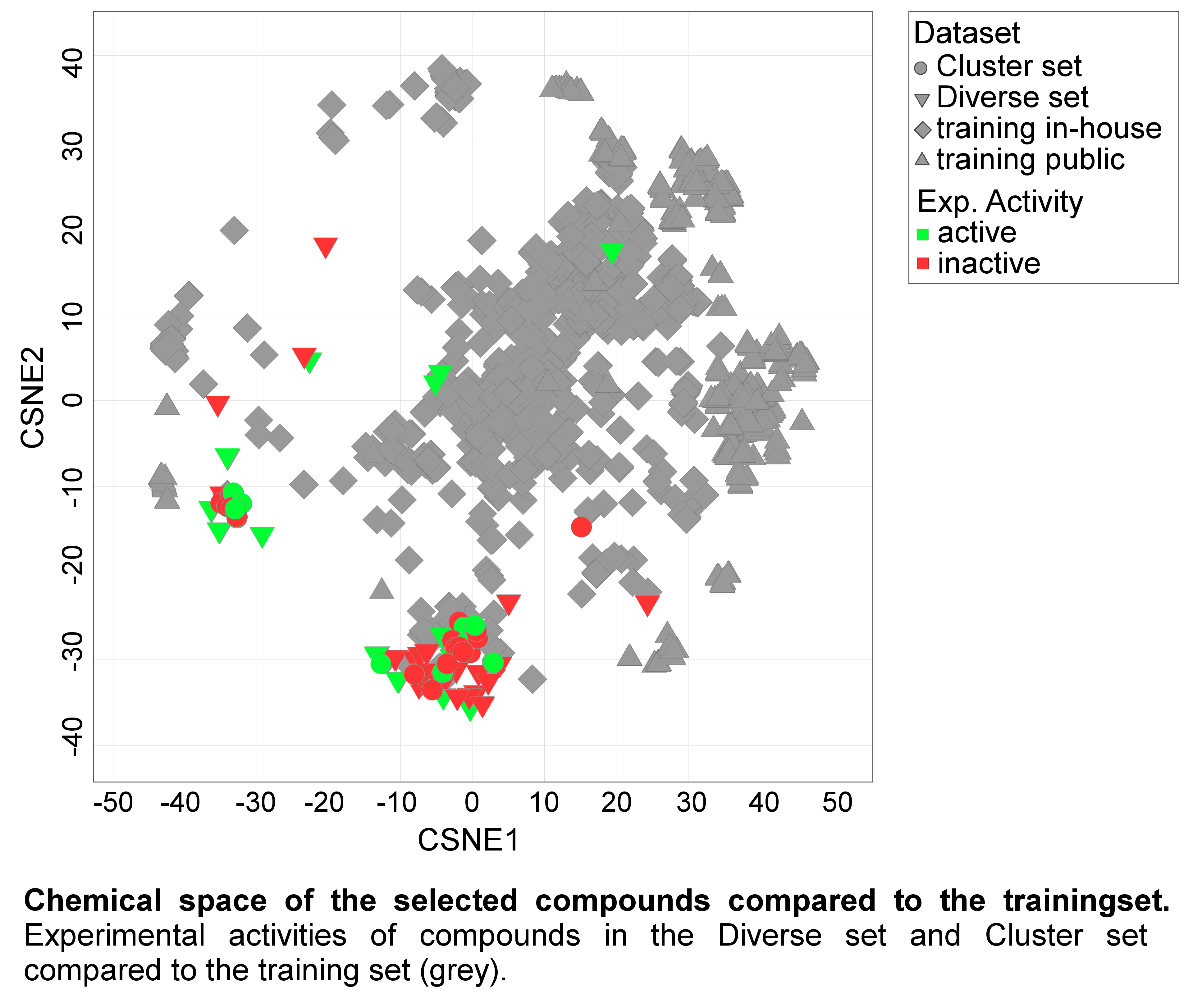

Supplement: Supplementary file 4 — Additional file 4. T-SNE representation of actives and inactives of selected compounds compared to the training set. [file 13321_2019_337_MOESM4_ESM.png]

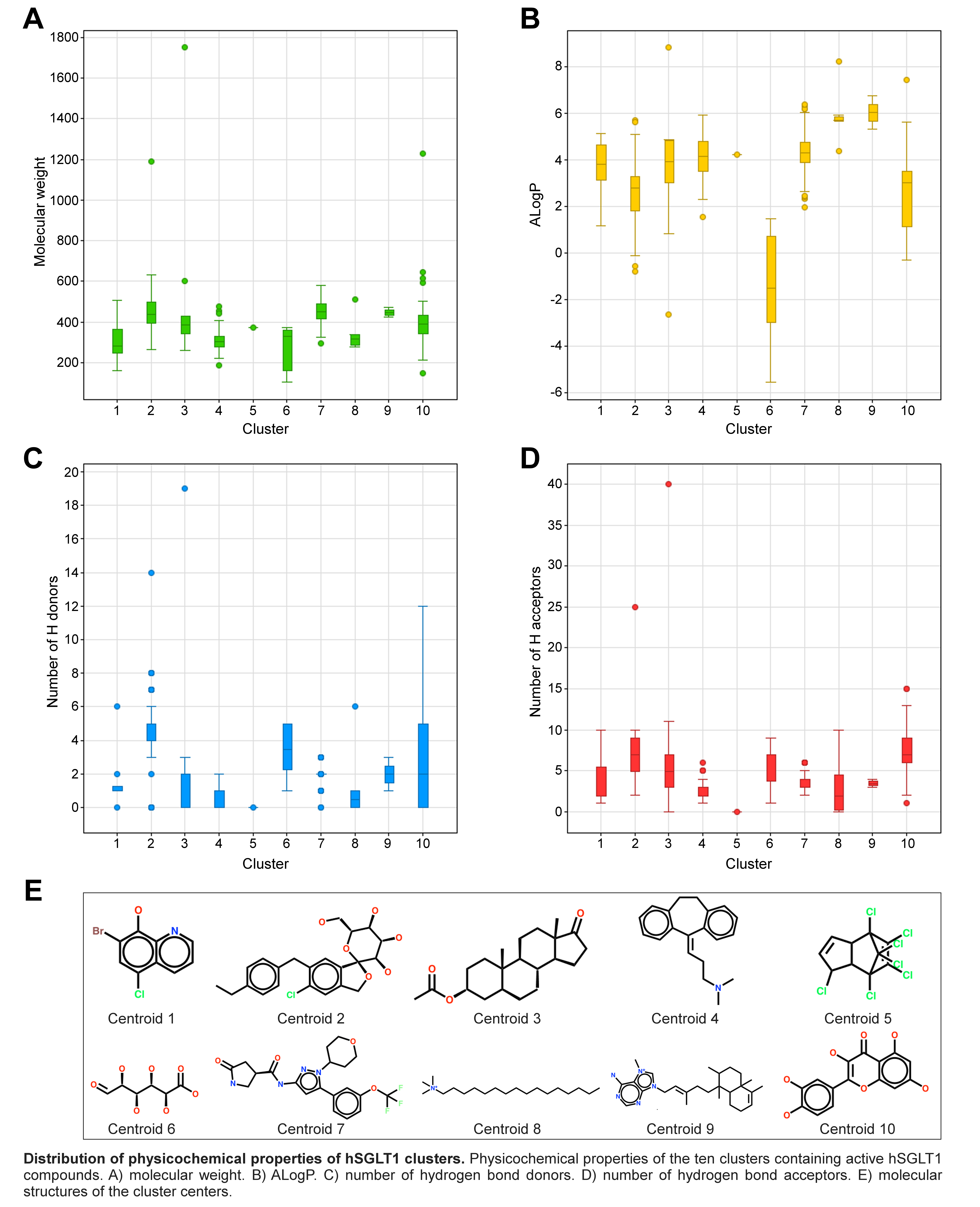

Supplement: Supplementary file 7 — Additional file 7. Cluster centers and distribution of physicochemical properties of hSGLT1 active compound clusters. [file 13321_2019_337_MOESM7_ESM.png]
