# Supplementary material for: Identification of novel small molecule inhibitors for solute carrier SGLT1 using proteochemometric modeling
Source: J Cheminform. 2019 Feb 14;11:15. doi: 10.1186/s13321-019-0337-8 (PMC6689890; doi:10.1186/s13321-019-0337-8)
Supplement: Supplementary file 2 — Additional file 2. Schematic overview of the experimental workflow of this study. [file 13321_2019_337_MOESM2_ESM.pdf]

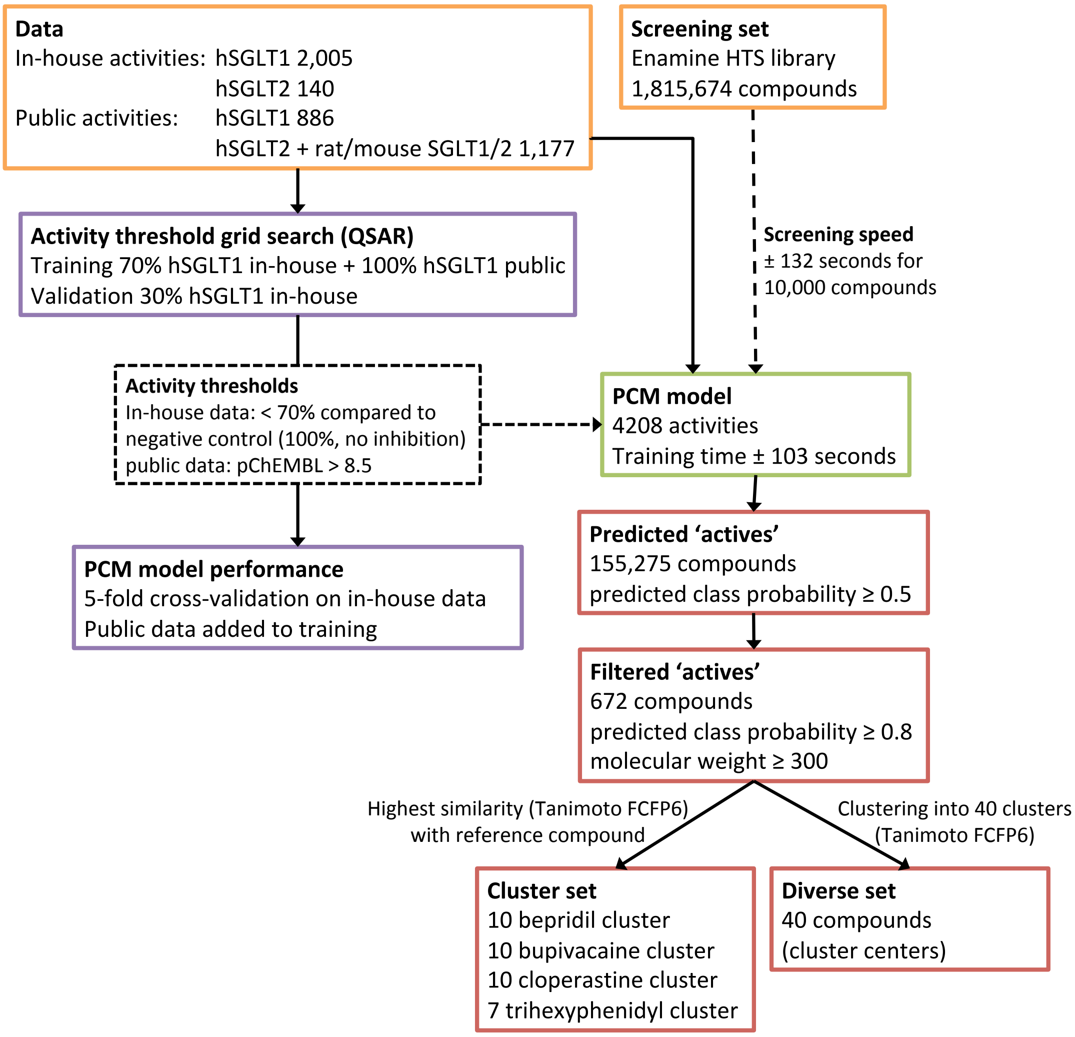


**Experimental workflow**. Datasets are indicated in orange boxes. Methods for model validation are in purple, the PCM model used in screening is in green, and compound sets derived from the Enamine screening set are colored red. The activity thresholds used in the final PCM model are derived by activity threshold grid searching and is indicated by the dotted-line box.
